# Distinct temporal trajectories of *EPAS1* mRNA expression during moderate-altitude training in Han and Tibetan endurance athletes

**DOI:** 10.3389/fcell.2026.1973797

**Published:** 2026-10-09

**Authors:** Qing-Qing Su, Hai-Lin Ma, Yan-Lin Sun, Fu-Hai Ma, Yan-Chang Shen, Jian Wang, Xiao-Wei Ma, Jiang-Long Zhang, Yang Zhang, Erik R. Swenson

**Affiliations:** 1 College of Physical Education, Qinghai Minzu University, Xining, China; 2 Institute of Sport Science in High Altitude Area, Qinghai Minzu University, Xining, China; 3 Plateau Brain Science Research Center, Tibet University, Lhasa, China; 4 Tibet Autonomous Region Key Laboratory for High Altitude Brain Science and Environmental Acclimatization, Tibet University, Lhasa, China; 5 Qinghai Institute of Sports Science, Xining, China; 6 School of Social Sports, Tianjin University of Sport, Tianjin, China; 7 Independent Person, Windermere, FL, United States; 8 Division of Pulmonary, Critical Care and Sleep Medicine, University of Washington, Seattle, WA, United States

**Keywords:** erythropoiesis, hemoglobin, HIF-2α, hypoxic stress, long noncoding RNA

## Abstract

**Purpose:**

*EPAS1* encodes hypoxia-inducible factor-2α and is strongly implicated in high-altitude adaptation, but its longitudinal transcriptional response during altitude training remains poorly understood. This study examined temporal changes in *EPAS1* mRNA expression during moderate-altitude training in Han and Tibetan endurance athletes.

**Methods:**

Han (n = 16) and Tibetan (n = 17) male endurance athletes completed 4 weeks of naturalistic training at approximately 2,260 m. Han athletes ascended from low altitude, whereas Tibetan athletes descended from habitual residence and training environments above 3,500 m. *EPAS1* mRNA expression was measured longitudinally, and whole-blood lncRNA and mRNA profiles were examined exploratorily in a subset of seven athletes per cohort.

**Results:**

*EPAS1* expression showed a significant group-by-time interaction (*p* < 0.001). In Han athletes, expression was 21.5% lower at Week 1 than at Baseline (expression ratio 0.785, 95% CI 0.681–0.906) and did not differ detectably from Baseline at Week 4 (0.997, 95% CI 0.864–1.150). In Tibetan athletes, expression was 28.5% higher at Week 1 (1.285, 95% CI 1.152–1.434) and remained 22.6% higher at Week 4 (1.226, 95% CI 1.099–1.368). No significant changes were detected in the measured hematological variables. Exploratory transcriptomic analysis showed differential expression by Week 1 in Han athletes and later-emerging whole-blood transcriptional changes in Tibetan athletes; no Gene Ontology Biological Process terms survived FDR correction.

**Conclusion:**

Han and Tibetan endurance athletes showed distinct temporal patterns of *EPAS1* mRNA expression during moderate-altitude training. These longitudinal findings provide a basis for further investigation of their physiological significance.

## 1 Introduction

Exposure to altitude reduces inspired oxygen availability and elicits physiological adjustments involving ventilation, cardiovascular function, and erythropoiesis (Moore, 2001; Simonson, 2015). These responses are coordinated partly through hypoxia-inducible factor (HIF) signaling, which regulates genes involved in oxygen transport, metabolism, and vascular function (Semenza, 2012). HIF-2α, encoded by endothelial PAS domain-containing protein 1 (*EPAS1*), is particularly relevant to human high-altitude adaptation. Genetic studies have identified *EPAS1* as a major target of natural selection in Tibetan highlanders, with adaptive variants associated with characteristic hematological responses to chronic hypoxia (Beall et al., 2010; Simonson et al., 2010; Yi et al., 2010). Although the genetic contribution of *EPAS1* to high-altitude adaptation is well established, its longitudinal transcriptional response during sustained physiological exposure remains less understood. Longitudinal changes in *EPAS1* mRNA therefore warrant direct investigation.

Endurance training at altitude provides a relevant setting in which to examine these dynamics. When repeated endurance training and environmental hypoxia occur together, as during an altitude-training camp, oxygen-responsive pathways are exposed to multiple physiological stimuli rather than reduced oxygen availability alone. Regulation of hypoxia-responsive genes may therefore vary over time as athletes adapt to the combined environmental and training demands. Altitude training may consequently provide a useful model for examining temporal *EPAS1* expression under sustained physiological stress.

Long noncoding RNAs (lncRNAs) may also contribute to transcriptional responses to physiological stress. lncRNA expression is responsive to hypoxia and exercise and can influence gene regulation through transcriptional and post-transcriptional mechanisms (Wohlwend et al., 2021; Li et al., 2022; Nickerson and Momen-Heravi, 2024). To our knowledge, their temporal behavior during sustained altitude training has not been characterized. lncRNA and mRNA profiling may therefore provide additional information on broader transcriptional changes accompanying the longitudinal *EPAS1* response.

Therefore, the primary objective of this study was to characterize changes in *EPAS1* mRNA expression during 4 weeks of naturalistic moderate-altitude training and to determine whether its temporal pattern differed between Han and Tibetan endurance athletes.

## 2 Methods

### 2.1 Experimental design

This observational longitudinal study followed endurance athletes during a 4-week naturalistic moderate-altitude training camp. Eligible athletes had no known cardiopulmonary, hematological, or metabolic disorders and no recent injuries that could affect training. Because no similar longitudinal altitude-training study of *EPAS1* expression was available to provide a defensible planning effect-size estimate, no *a priori* sample size calculation was performed. Accordingly, the study was considered exploratory, and effect estimates are presented with 95% confidence intervals to emphasize their precision. Of the 83 athletes attending the camp (45 Han and 38 Tibetan), 33 agreed to participate before departure. No female athletes volunteered. Participant characteristics are presented in Table 1.

**TABLE 1:** Participant characteristics.

Variable	Han (n = 16)	Tibetan (n = 17)
Age, year	19.5 ± 3.2	18.4 ± 3.5
Height, cm	176 ± 6	170 ± 5
Body weight, kg	63.2 ± 6.0	57.3 ± 6.4
Body mass index, kg/m^2^	20.4 ± 1.3	19.8 ± 1.8
VO_2max_, mL·kg^-1^·min^-1^	55.6 ± 3.8	56.7 ± 3.8
Training experience, years	4.8 ± 1.8	4.2 ± 1.2
Main event, n (%)		
5,000 m	10 (62.5%)	12 (70.6%)
Race walking	6 (37.5%)	5 (29.4%)
Competitive level, n (%)		
National master sportsman	2 (12.5%)	3 (17.6%)
National first-class athlete	6 (37.5%)	4 (23.5%)
National second-class athlete	8 (50%)	2 (11.8%)
No national classification	—	8 (47.1%)
Habitual residence and training altitude, m	<500	3,500–3,900

Continuous variables are presented as mean ± standard deviation, and categorical variables as n (%).

Training was conducted at the Duoba Highland Training Base in Qinghai Province at approximately 2,260 m. Athletes followed coach-directed programs that were not standardized by the research team. Training distance was recorded using Polar devices. Mean cumulative distance per athlete during the 4-week camp was 824 km in the Han group and 812 km in the Tibetan group. No acute illness was reported during the study. Iron status, dietary intake, iron supplementation, and medication use were not systematically recorded.

The study was approved by the Institutional Review Board of Qinghai Minzu University (approval number: 2020–015) and conducted in accordance with the Declaration of Helsinki. All participants provided written informed consent. For participants younger than 18 years, parental or guardian consent and participant assent were obtained.

### 2.2 Measurement schedule

Demographic and sporting characteristics, including pre-departure maximum oxygen uptake measured at each athlete’s habitual training site, were obtained from official team records. Study measurements were obtained at Baseline, Week 1, and Week 4. Han baseline assessments were conducted at approximately 100 m 3 days before travel to the training camp. Tibetan athletes traveled from their respective locations in Tibet to Lhasa, assembled there, and then flew together to Xining. Because suitable research equipment and cold-storage facilities were unavailable at their habitual training sites, baseline measurements could not be obtained before descent. After landing, the athletes traveled for approximately 1 h by bus to the training base and completed baseline assessments at 2,260 m within 6 h of arrival in Xining. Week one and Week four assessments were conducted on Monday mornings after each completed training week. According to the coach-provided training schedules, Sunday afternoon was a scheduled rest period for both groups before these assessments.

### 2.3 Physiological and hematological measurements

Resting heart rate and peripheral oxygen saturation were measured after 10 min of seated rest using a fingertip pulse oximeter (Prince-100NW, Heal Force, China). Venous blood samples were gently inverted 3–5 times and analyzed immediately using an automated hematology analyzer (Mindray BC-5180 CRP, Shenzhen, China).

### 2.4 Serum RNA extraction and quantification of *EPAS1* expression

Serum samples were analyzed by an external laboratory (CapitalBio Technology Co., Ltd., China). Total RNA was extracted using TRIzol LS. *EPAS1* mRNA was quantified using SYBR Green real-time PCR with the following primers: forward, 5′-GGG​ACT​TAC​ACA​GGT​GGA​GC-3′; reverse, 5′-CGA​ATC​TCC​TCA​TGG​TCG​CA-3′. 18S rRNA was used as the housekeeping gene, and reactions were performed in technical triplicate. The laboratory reported that the completed reactions met its internal quality-control requirements. For each sample, the change in cycle threshold (Δ*Ct*) was defined as *Ct*_*EPAS1*_ – *Ct*_18S_, and normalized expression was calculated as 2^−Δ*Ct*^.

### 2.5 Whole-blood lncRNA and mRNA microarray profiling and exploratory functional analysis

Due to funding constraints, seven athletes from each cohort were selected for exploratory transcriptomic analysis. The subset was not randomly selected; selection was restricted to athletes classified as National Master Sportsman or National First-Class Athlete with complete longitudinal samples available at Baseline, Week 1, and Week 4. Samples were stored at −80 °C and analyzed by CapitalBio Technology Co., Ltd (Beijing, China). Total RNA was extracted using PAXgene. RNA purity was assessed by A260/280, RNA quantity by Qubit, and integrity by denaturing gel electrophoresis. The laboratory quality criterion for purity was A260/280 ≥ 1.70, and RNA integrity was considered acceptable when the 28S:18S rRNA band intensity was approximately 1:1 or greater. Genome-wide lncRNA and mRNA profiling was performed using the CapitalBio Human LncRNA Array v4 (4 × 180K). RNA labeling, hybridization, scanning, and primary data processing were performed by the external laboratory according to its standardized microarray protocol. The laboratory reported that all analyzed samples met its quality-control requirements.

Raw probe-level intensities were background corrected, quantile normalized, log_2_ transformed, and filtered for low-intensity transcripts in GeneSpring GX 13.0. Differential-expression analyses were subsequently performed in R using the *limma* package (Ritchie et al., 2015). Analyses were conducted separately within the Han and Tibetan cohorts. For each transcript type, the design matrix included participant and time, thereby accounting for the repeated measurements obtained from the same seven athletes. Empirical-Bayes moderated linear models were used to estimate three prespecified contrasts: Week 1 versus Baseline, Week 4 versus Baseline, and Week 4 versus Week 1. Positive log_2_ fold-change values indicate higher expression at the later time point. Differential expression was defined at the probe level as |log_2_ fold change| ≥1 together with a Benjamini–Hochberg false discovery rate (FDR)-adjusted *p* value ≤0.05. Given the seven participants per cohort, these analyses were considered exploratory; empirical-Bayes moderation was used to stabilize probe-wise variance estimates, although statistical power to detect smaller expression changes remained limited.

For biological annotation, differentially expressed mRNA probes with valid Entrez Gene identifiers were collapsed to unique genes. When multiple significant probes mapped to the same Entrez Gene identifier, the gene was counted once; directional classification was assigned when the significant probes were concordant, whereas genes represented by significant probes with opposing directions were classified as discordant. Differentially expressed lncRNA probes were similarly mapped to unique annotated lncRNA transcript identifiers. Probe-level results were retained for statistical reporting, whereas gene- and transcript-level identifiers were used for biological interpretation.

Gene Ontology (GO) Biological Process enrichment was performed using the *clusterProfiler* package (Wu et al., 2021) on unique Entrez-annotated differentially expressed genes. The enrichment background comprised all unique Entrez-annotated mRNA genes tested on the array within the corresponding cohort. Benjamini–Hochberg adjustment was applied to GO enrichment tests, with FDR ≤0.05 considered significant.

### 2.6 Statistical analysis

*EPAS1* expression was analyzed on the Δ*Ct* scale. Because the external laboratory provided participant-level normalized expression as 2^−ΔΔCT^, Δ*Ct* values were recovered using the exact inverse transformation, −log_2_ (2^−Δ*Ct*^). The primary linear mixed-effects model included group, time, and the group-by-time interaction as fixed effects and participant as a random intercept. Degrees of freedom were estimated using the Satterthwaite method. Within each cohort, changes from Baseline to Week 1 and Week 4 were estimated with 95% confidence intervals (CIs). Between-group differences in these temporal changes were also estimated with 95% CIs. For interpretability, within-group contrasts were back-transformed as 2^−ΔΔ*Ct*^ and reported as follow-up-to-Baseline expression ratios. Between-group contrasts were back-transformed and reported as ratios of expression changes (Tibetan/Han), with values >1 indicating a greater relative increase, or a less pronounced decrease, in Tibetan than Han athletes. Holm adjustment was applied separately to the two within-group contrasts in each cohort and to the two between-group contrasts.

To examine the robustness of the *EPAS1* findings to available participant characteristics, sensitivity analyses were conducted separately for age, body mass index, VO_2_max, years of training experience, and competitive level. Continuous covariates were mean-centered, and each covariate and its interaction with time were added separately to the primary group-by-time model to avoid overparameterization. Competitive level was entered as a categorical variable. Each sensitivity analysis included participants with available data for the corresponding covariate, without imputation, and adjusted estimates were compared with unadjusted models fitted to the same complete-case subset. Because the random-intercept variance in the primary *EPAS1* model was estimated at the boundary, a fixed-effects model with the same group-by-time specification was also examined as a sensitivity analysis.

For physiological and hematological variables, group, time, and group-by-time interaction effects were assessed using linear mixed-effects models with participant as a random intercept. Within-group temporal changes were further assessed using repeated-measures analysis of variance when distributional assumptions were satisfied and the Friedman test otherwise. Significant omnibus tests were followed by paired comparisons with Holm adjustment.

All statistical analyses were performed in R 4.6.0. Tests were two-sided, with *p* < 0.05 considered statistically significant. Available observations were analyzed without imputation. Microarray expression data are available from the NCBI Gene Expression Omnibus under accession GSE333506. Participant-level normalized expression data used for the transcriptomic analyses are deposited in Figshare (DOI: 10.6084/m9.figshare.31151641.v2).

## 3 Results

### 3.1 Physiological and hematological responses

Physiological and hematological measures at Baseline, Week 1, and Week 4 are presented in Table 2. No significant group-by-time interactions were observed for the measured variables. Within-group analyses likewise showed no significant changes across the 4-week training period. Resting heart rate and peripheral oxygen saturation remained relatively stable, as did hemoglobin concentration, red blood cell count, hematocrit, and red-cell indices.

**TABLE 2:** Physiological responses of Han and Tibetan endurance athletes during 4 weeks of altitude training.

Variable	Han (n = 16)			Tibetan (n = 17)		
	Baseline	Week 1	Week 4	Baseline	Week 1	Week 4
RHR, bpm	58 ± 6	63 ± 6	63 ± 8	55 ± 8	62 ± 13	55 ± 10
SpO_2_, %	94.3 ± 1.7	95.1 ± 2.1	94.5 ± 2.2	95.4 ± 1.5	95.9 ± 1.7	95.1 ± 1.5
HGB, g/L	154.4 ± 8.8	152.4 ± 8.1	154.5 ± 8.3	155.4 ± 11.0	152.5 ± 13.9	152.2 ± 13.6
RBC, ×10^12^/L	5.3 ± 0.3	5.2 ± 0.3	5.3 ± 0.3	5.4 ± 0.3	5.3 ± 0.3	5.3 ± 0.3
MCV, fL	87.1 ± 3.1	87.0 ± 3.2	87.4 ± 3.5	85.3 ± 3.8	85.3 ± 3.8	85.7 ± 4.0
HCT, %	46.4 ± 2.5	45.6 ± 3.0	46.5 ± 2.7	46.5 ± 3.5	45.7 ± 4.4	45.5 ± 3.9
MCH, pg	29.0 ± 1.2	29.7 ± 2.2	29.1 ± 1.6	28.6 ± 1.4	28.5 ± 1.3	29.2 ± 3.1
MCHC, g/L	333 ± 7	334 ± 10	333 ± 8	335 ± 7	334 ± 4	334 ± 7

Values are expressed as mean ± standard deviation. HCT, hematocrit; HGB, hemoglobin; MCH, mean corpuscular hemoglobin; MCHC, mean corpuscular hemoglobin concentration; MCV, mean corpuscular volume; SpO_2_ = peripheral oxygen saturation; RHR, resting heart rate; RBC, red blood cell count.

### 3.2 *EPAS1* expression dynamics

Longitudinal *EPAS1* expression is shown in Figure 1. The linear mixed-effects model demonstrated a significant group-by-time interaction (*F* (2,75) = 15.25; *p* < 0.001; Table 3). In Han athletes, *EPAS1* expression was 21.5% lower at Week 1 than at Baseline (expression ratio 0.785, 95% CI 0.681–0.906), whereas expression at Week 4 did not differ detectably from Baseline (0.997, 95% CI 0.864–1.150). In Tibetan athletes, expression was 28.5% higher at Week 1 (1.285, 95% CI 1.152–1.434) and remained 22.6% higher at Week 4 (1.226, 95% CI 1.099–1.368).

**FIGURE 1:**
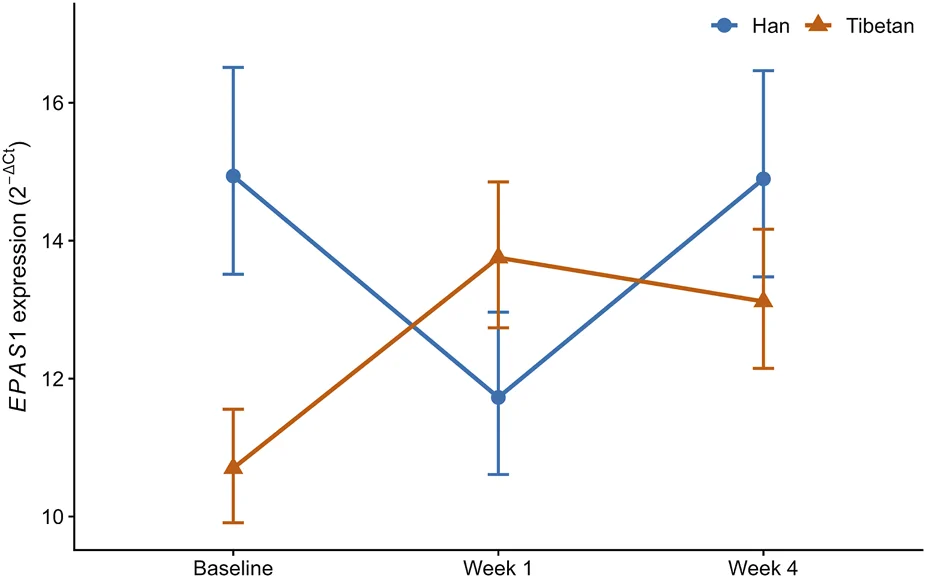
Temporal changes in *EPAS1* mRNA expression during 4 weeks of moderate-altitude training in Han and Tibetan endurance athletes. Values are normalized expression (2^−Δ*Ct*^) with 95% confidence intervals.

**TABLE 3:** Temporal changes in *EPAS1* mRNA expression and between-group differences in change.

Contrast	Expression estimate (95% CI)	Change, %	*p* value
Within-group changes			
Han: Week 1 vs. baseline	0.785 (0.681–0.906)	−21.5	0.003
Han: Week 4 vs. baseline	0.997 (0.864–1.150)	−0.3	0.969
Tibetan: Week 1 vs. baseline	1.285 (1.152–1.434)	+28.5	<0.001
Tibetan: Week 4 vs. baseline	1.226 (1.099–1.368)	+22.6	<0.001
Between-group differences in change			
Week 1 vs. baseline	1.637 (1.367–1.960)	—	<0.001
Week 4 vs. baseline	1.229 (1.027–1.472)	—	0.025

*EPAS1* expression was analyzed on the Δ*Ct* (*Ct*_*EPAS1*_ – *Ct*_18S_) scale using a linear mixed-effects model with group, time, and their interaction as fixed effects and participant as a random intercept. Estimates are presented on the back-transformed expression scale for interpretability. For within-group contrasts, values are follow-up/Baseline expression ratios; values > 1 indicate higher expression and values < 1 indicate lower expression at follow-up. For between-group contrasts, values are ratios of expression changes (Tibetan/Han); values > 1 indicate a greater relative increase, or a less pronounced decrease, in Tibetan than Han athletes. For example, at Week 1 the expression ratios were 1.285 in Tibetan and 0.785 in Han athletes, giving a Tibetan/Han ratio of 1.285/0.785 = 1.637. *P* values were adjusted using the Holm method within each family of contrasts.

The between-group difference in temporal change was greatest at Week 1, when the Tibetan/Han ratio of expression changes was 1.637 (95% CI 1.367–1.960), and remained evident at Week 4 (1.229, 95% CI 1.027–1.472). Sensitivity analyses separately accounting for age, body mass index, VO_2_max, training experience, and competitive level did not materially alter the group-by-time pattern. The fixed-effects sensitivity analysis likewise produced materially unchanged estimates.

### 3.3 Exploratory whole-blood transcriptomic profiles

Paired differential-expression analysis revealed distinct temporal patterns between the two cohorts (Table 4). In Han athletes, differential expression was already evident at Week 1 and remained extensive at Week 4 relative to Baseline, with predominantly lower expression at both time points. However, direct comparison of Weeks 4 and 1 identified only four differentially expressed mRNA probes, suggesting comparatively limited additional mRNA-level change after the initial response. Changes in lncRNA expression between Weeks 1 and 4 were more apparent.

**TABLE 4:** Exploratory differential expression of whole-blood mRNA and lncRNA across altitude-training time points.

Cohort	Contrast	DE mRNA probes	Unique DE mRNA genes			DE lncRNA probes	Unique lncRNA transcripts	
			Up	Down	Discordant		Up	Down
Han	W1 vs. W0	202	6	162	0	113	4	109
Han	W4 vs. W0	420	123	243	0	789	146	643
Han	W4 vs. W1	4	0	3	0	72	11	61
Tibetan	W1 vs. W0	0	0	0	0	0	0	0
Tibetan	W4 vs. W0	755	479	225	0	1,071	322	749
Tibetan	W4 vs. W1	427	254	132	1	508	151	357

Differential expression (DE) was defined at the probe level as |log_2_ fold change| ≥ 1 and Benjamini–Hochberg FDR-adjusted *p* ≤ .05 using paired *limma* models that included participant and time. Up- and downregulation refer to the later time point relative to the earlier time point. Unique DE mRNA genes were defined by valid Entrez Gene identifiers; therefore, gene counts may be lower than DE probe counts. Unique lncRNA transcripts were defined by annotated lncRNA transcript identifiers. W0, baseline; W1, Week 1; W4, Week 4.

In Tibetan athletes, no mRNA or lncRNA probes met the differential-expression criteria at Week 1 relative to Baseline. In contrast, extensive differential expression was present at Week 4, and direct comparison of Weeks 4 and 1 identified 427 mRNA probes and 508 lncRNA probes. This pattern is consistent with a later-emerging whole-blood transcriptional response during the 4-week training period.

GO Biological Process enrichment of the overall differentially expressed gene sets did not identify any terms that survived FDR correction.

## 4 Discussion

The principal finding of this study was a distinct temporal pattern of *EPAS1* mRNA expression between Han and Tibetan male endurance athletes during 4 weeks of moderate-altitude training. In Han athletes, expression was lower at Week 1 and was no longer detectably different from Baseline at Week 4. Tibetan athletes showed higher expression at Week 1 that remained elevated at Week 4. The between-group divergence was greatest at Week 1 but remained evident at Week 4. By comparison, the measured erythrocyte-related indices showed no significant group-by-time effects or within-group changes.

These findings concern *EPAS1* transcript abundance and do not directly indicate changes in HIF-2α protein abundance or activity. Although *EPAS1* encodes HIF-2α, HIF signaling is regulated substantially through oxygen-dependent stabilization and degradation of HIF-α proteins (Schofield and Ratcliffe, 2004; Choudhry and Harris, 2018). Changes in *EPAS1* mRNA therefore need not produce proportional changes in HIF-2α activity or downstream transcription. HIF-2α protein, erythropoietin (EPO), and established downstream HIF targets were not measured. The observed trajectories should therefore be interpreted as longitudinal changes in *EPAS1* mRNA expression rather than evidence of altered HIF-2α signaling.

*EPAS1* is one of the strongest genetic targets of high-altitude adaptation identified in Tibetan highlanders (Beall et al., 2010; Simonson et al., 2010; Yi et al., 2010). Functional evidence shows that Tibetan-enriched *EPAS1* variants are associated with reduced transcription under chronic hypoxic conditions, including lower expression in endothelial cells carrying the adaptive haplotype during prolonged hypoxia and in Tibetan placental tissue (Peng et al., 2017). In this context, the increase in *EPAS1* expression after the Tibetan athletes descended from habitual environments above 3,500 m to 2,260 m is of interest. One hypothesis is that reduced hypoxic exposure permitted a gradual shift away from the transcriptional state associated with chronic high-altitude residence. The present design, however, cannot establish such a re-equilibration process. The Tibetan Baseline sample was obtained within several hours of arrival at 2,260 m, after descent and travel had already occurred, and no sample was collected at habitual altitude immediately before descent. The Baseline-to-Week 1-to-Week 4 trajectory may therefore reflect a response already underway before Baseline, effects associated with descent and travel, or other temporal processes. Future studies incorporating pre-descent sampling at habitual altitude will be needed to distinguish among these possibilities.

The Han trajectory occurred under a different altitude transition. These athletes moved from low to moderate altitude and showed an early reduction in *EPAS1* expression, with no detectable difference from Baseline by Week 4. Evidence on *EPAS1* transcription during endurance exercise remains limited. Higher skeletal-muscle *EPAS1* expression has been observed in endurance than power athletes, together with an overall increase across aerobic-training transcriptomic datasets (Dautova et al., 2026). Although skeletal-muscle findings cannot be directly compared with circulating RNA, they support the broader view that *EPAS1* transcription is dynamic. The present study cannot determine whether the Han trajectory was driven primarily by hypoxic exposure, endurance training, their interaction, or other features of the training camp.

Resting peripheral oxygen saturation remained relatively stable throughout the study, suggesting that the sustained systemic hypoxemic stimulus detectable under the measurement conditions was modest. This may partly explain the lack of detectable changes in hemoglobin concentration, hematocrit, and red blood cell count. Altitude-training studies have likewise reported heterogeneous hematological responses (McLean et al., 2013; Płoszczyca et al., 2018; Nummela et al., 2021). However, these concentration-based measures cannot determine whether total red-cell volume or hemoglobin mass changed, and EPO was not measured. The present findings therefore indicate an absence of detectable change in the measured hematological variables but do not exclude an early, transient, or otherwise unmeasured erythropoietic response.

Exploratory whole-blood transcriptomic analysis also suggested different temporal patterns between cohorts. Han athletes showed differential expression by Week 1 with little additional mRNA-level change between Weeks 1 and 4, whereas Tibetan athletes showed no significant differential expression at Week 1 but extensive changes by Week 4, including substantial direct Week 1–4 differences. This pattern is consistent with later-emerging transcriptional changes in the Tibetan cohort. With only seven participants per cohort, however, these findings remain descriptive. No GO Biological Process terms survived FDR correction for the overall differentially expressed gene sets, and the results do not establish specific adaptive mechanisms, pathways, or tissue-specific responses. Changes in whole-blood cellular composition may also have contributed.

Several limitations warrant emphasis. The study was exploratory, included a relatively small male-only sample, and was conducted under naturalistic rather than experimentally standardized training conditions. Most importantly, Baseline timing and altitude exposure differed fundamentally between cohorts. The central group-by-time interaction therefore reflects both cohort and differing altitude-transition and Baseline-measurement conditions, limiting attribution of the trajectories to ethnicity, genetic background, or altitude-transition direction. Iron status, dietary intake, iron supplementation, and medication use were not systematically recorded and may have influenced erythropoietic physiology or *EPAS1*-related responses. Differential leukocyte counts were unavailable, and HIF-2α protein, EPO, reticulocytes, blood volume, and hemoglobin mass were not measured. The transcriptomic subset was also small. Together, these limitations constrain mechanistic inference and generalizability.

In conclusion, Han and Tibetan male endurance athletes showed distinct temporal patterns of *EPAS1* mRNA expression during 4 weeks of moderate-altitude training. The Tibetan trajectory after descent from habitual high altitude raises testable questions regarding transcriptional responses to reduced hypoxic exposure, while the Han trajectory indicates a different early response following ascent from low altitude. Future studies incorporating pre-transition sampling, HIF-2α protein and downstream signaling measurements, erythropoietic markers, hemoglobin mass, and cell-specific transcriptomic approaches will be needed to establish the physiological and mechanistic significance of these observations.

## Data Availability

The datasets presented in this study can be found in online repositories. The names of the repository/repositories and accession number(s) can be found below: https://www.ncbi.nlm.nih.gov/, GSE333506.

## References

[B1] Beall C. M. Cavalleri G. L. Deng L. Elston R. C. Gao Y. Knight J. (2010). Natural selection on EPAS1 (HIF2α) associated with low hemoglobin concentration in Tibetan highlanders. Proc. Natl. Acad. Sci. 107 (25), 11459–11464. 10.1073/pnas.1002443107 20534544 PMC2895075

[B2] Choudhry H. Harris A. L. (2018). Advances in hypoxia-inducible factor biology. Cell Metab. 27 (2), 281–298. 10.1016/j.cmet.2017.10.005 29129785

[B3] Dautova A. Z. Valeeva E. V. Semenova E. A. Mavliev F. A. Zverev A. A. Nazarenko A. S. (2026). EPAS1 is associated with human muscle fiber composition and endurance phenotypes. Physiol. Rep. 14 (10), e70928. 10.14814/phy2.70928 42157436 PMC13423621

[B4] Li H. Trager L. E. Liu X. Hastings M. H. Xiao C. Guerra J. (2022). lncExACT1 and DCHS2 regulate physiological and pathological cardiac growth. Circulation 145 (16), 1218–1233. 10.1161/CIRCULATIONAHA.121.056850 35114812 PMC9056949

[B5] McLean B. D. Buttifant D. Gore C. J. White K. Kemp J. (2013). Year-to-year variability in haemoglobin mass response to two altitude training camps. Br. J. Sports Med. 47 (Suppl. 1), i51–i58. 10.1136/bjsports-2013-092744 24282208 PMC3903153

[B6] Moore L. G. (2001). Human genetic adaptation to high altitude. High Alt. Med. and Biol. 2 (2), 257–279. 10.1089/152702901750265341 11443005

[B7] Nickerson J. A. Momen-Heravi F. (2024). Long non-coding RNAs: roles in cellular stress responses and epigenetic mechanisms regulating chromatin. Nucleus 15 (1), 2350180. 10.1080/19491034.2024.2350180 38773934 PMC11123517

[B8] Nummela A. Eronen T. Koponen A. Tikkanen H. Peltonen J. E. (2021). Variability in hemoglobin mass response to altitude training camps. Scand. J. Med. and Sci. Sports 31 (1), 44–51. 10.1111/sms.13804 32783231

[B9] Peng Y. Cui C. He Y. Ouzhuluobu O. Zhang H. Yang D. (2017). Down-regulation of EPAS1 transcription and genetic adaptation of tibetans to high-altitude hypoxia. Mol. Biol. Evol. 34 (4), 818–830. 10.1093/molbev/msw280 28096303 PMC5400376

[B10] Płoszczyca K. Langfort J. Czuba M. (2018). The effects of altitude training on erythropoietic response and hematological variables in adult athletes: a narrative review. Front. Physiology 9, 375. 10.3389/fphys.2018.00375 29695978 PMC5904371

[B11] Ritchie M. E. Phipson B. Wu D. Hu Y. Law C. W. Shi W. (2015). Limma powers differential expression analyses for RNA-Sequencing and microarray studies. Nucleic Acids Res. 43 (7), e47. 10.1093/nar/gkv007 25605792 PMC4402510

[B12] Schofield C. J. Ratcliffe P. J. (2004). Oxygen sensing by HIF hydroxylases. Nat. Rev. Mol. Cell Biol. 5 (5), 343–354. 10.1038/nrm1366 15122348

[B13] Semenza G. L. (2012). Hypoxia-inducible factors in physiology and medicine. Cell 148 (3), 399–408. 10.1016/j.cell.2012.01.021 22304911 PMC3437543

[B14] Simonson T. S. (2015). Altitude adaptation: a glimpse through various lenses. High Alt. Med. and Biol. 16 (2), 125–137. 10.1089/ham.2015.0033 26070057 PMC4490743

[B15] Simonson T. S. Yang Y. Huff C. D. Yun H. Qin G. Witherspoon D. J. (2010). Genetic evidence for high-altitude adaptation in Tibet. Science 329 (5987), 72–75. 10.1126/science.1189406 20466884

[B16] Wohlwend M. Laurila P.-P. Williams K. Romani M. Lima T. Pattawaran P. (2021). The exercise-induced long noncoding RNA CYTOR promotes fast-twitch myogenesis in aging. Sci. Transl. Med. 13 (623), eabc7367. 10.1126/scitranslmed.abc7367 34878822

[B17] Wu T. Hu E. Xu S. Chen M. Guo P. Dai Z. (2021). clusterProfiler 4.0: a universal enrichment tool for interpreting omics data. Innovation 2 (3), 100141. 10.1016/j.xinn.2021.100141 34557778 PMC8454663

[B18] Yi X. Liang Y. Huerta-Sanchez E. Jin X. Cuo Z. X. P. Pool J. E. (2010). Sequencing of 50 human exomes reveals adaptation to high altitude. Science 329 (5987), 75–78. 10.1126/science.1190371 20595611 PMC3711608

